# Plasma metabolomic profiling of proliferative diabetic retinopathy

**DOI:** 10.1186/s12986-019-0358-3

**Published:** 2019-05-28

**Authors:** Xiao-Rong Zhu, Fang-yuan Yang, Jing Lu, Hui-rong Zhang, Ran Sun, Jian-Bo Zhou, Jin-Kui Yang

**Affiliations:** 1 Department of Endocrinology, Beijing Tongren Hospital, Capital Medical University, Beijing, 100073 China; 2 Beijing Key Laboratory of Diabetes Research and Care, Beijing, China; 3 Beijing Diabetes Institute, Beijing, China

**Keywords:** Type 2 diabetes, Proliferative diabetic retinopathy, Metabolomics, Metabolic profiling, LC-MS

## Abstract

**Background:**

Proliferative diabetic retinopathy (PDR), a sight-threatening retinopathy, is the leading cause of irreversible blindness in adults. Despite strict control of systemic risk factors, a fraction of patients with diabetes develop PDR, suggesting the existence of other potential pathogenic factors underlying PDR. This study aimed to investigate the plasma metabotype of patients with PDR and to identify novel metabolite markers for PDR. Biomarkers identified from this study will provide scientific insight and new strategies for the early diagnosis and intervention of diabetic retinopathy.

**Methods:**

A total of 1024 patients with type 2 diabetes were screened. To match clinical parameters between case and control subjects, patients with PDR (PDR, *n* = 21) or those with a duration of diabetes of ≥10 years but without diabetic retinopathy (NDR, *n* = 21) were assigned to the present case-control study. Distinct metabolite profiles of serum were examined using liquid chromatography-mass spectrometry (LC-MS).

**Results:**

The distinct metabolites between PDR and NDR groups were significantly enriched in 9 KEGG pathways (*P* < 0.05, impact > 0.1), namely, alanine, aspartate and glutamate metabolism, caffeine metabolism, beta-alanine metabolism, purine metabolism, cysteine and methionine metabolism, sulfur metabolism, sphingosine metabolism, and arginine and proline metabolism. A total of 63 altered metabolites played important roles in these pathways. Finally, 4 metabolites were selected as candidate biomarkers for PDR, namely, fumaric acid, uridine, acetic acid, and cytidine. The area under the curve for these biomarkers were 0.96, 0.95, 1.0, and 0.95, respectively.

**Conclusions:**

This study suggested that impairment in the metabolism of pyrimidines, arginine and proline were identified as metabolic dysregulation associated with PDR. And fumaric acid, uridine, acetic acid, and cytidine might be potential biomarkers for PDR. Fumaric acid was firstly reported as a novel metabolite marker with no prior reports of association with diabetes or diabetic retinopathy, which might provide insights into potential new pathogenic pathways for diabetic retinopathy.

**Electronic supplementary material:**

The online version of this article (10.1186/s12986-019-0358-3) contains supplementary material, which is available to authorized users.

## Introduction

Diabetic retinopathy (DR) is one of the most common microvascular complications associated with diabetes and the leading cause of irreversible blindness in adults worldwide [1]. The number of diabetic patients in China ranks first in the world, and more than 1.6 million are legally blind due to diabetic retinopathy [2]. Therefore, there is an urgent need in China for early detection and intervention with respect to this clinically significant disease. Current studies suggest that glucose level and duration of disease are major systemic risk factors for the development and progression of microvascular complications, including DR [3, 4]. However, these risk factors could not explain the great variability that characterizes the evolution and rate of progression of the retinopathy in different diabetic patients, suggesting other factors may also involve [5]. It is a fact that many patients with intensive control of glycemic continue to develop DR, while patients with poor glycemic control may not progress DR. There is increasing evidence to suggest that “metabolic memory” might contribute to these different developmental phenotypes of DR [6]. The term metabolic memory refers to continual epigenetic modification caused by improper glycemic control in the early stage of diabetes, such individuals continue to progress diabetes complications even after glycemic control reaches the normal range for a period of time [7]. The mechanism of metabolic memory may as a result of influences of gene–environment interactions [7]. Detection of epigenetic signatures in DR could be valuable for timely diagnosis and prompt treatment to prevent progression of the disease [8]. Therefore, the discovery of biomarkers that lead to variations in the progression of DR thus become essential as these biomarkers will provide insight on the pathogenic pathways that are currently unknown and may serve as new strategies for the early diagnosis and intervention of diabetic retinopathy [4].

Metabolomics, a newly discovered “omics” field, detects the overall and dynamic changes of whole endogenous metabolites in organisms including nucleic acids, proteins, lipids and other small molecules [9]. It is currently recognized as a very powerful tool that is complementary to genomic, transcriptomic, or proteomic data. In the past decades, metabolomics has been increasingly used to identify biomarkers associated with metabolic diseases [10]. DR is a complex metabolic disease relates to the interplay of genetic and environmental factors [11], thus the discovery of distinct metabolic signature of DR and the associated pathways could help improve our understanding of the pathophysiology and mechanisms of disease. Barba et al. [12] identified metabolite markers of DR in the vitreous humor. However, the invasiveness of vitreous sampling limits the potential for study replication and clinical translation of any biomarkers identified from vitreous fluid. In contrast, plasma or serum remains the choice of metabolic fluid [6]. In the present study, we aimed to investigate the plasma metabotype of proliferative DR (PDR) (a sight-threatening retinopathy), and to identify novel metabolite markers of PDR. The investigation of metabolite markers of DR will be helpful to explore the mechanism of the occurrence and progression of DR in different stages of disease.

## Materials and methods

### Sample selection

**Fig. 1:**
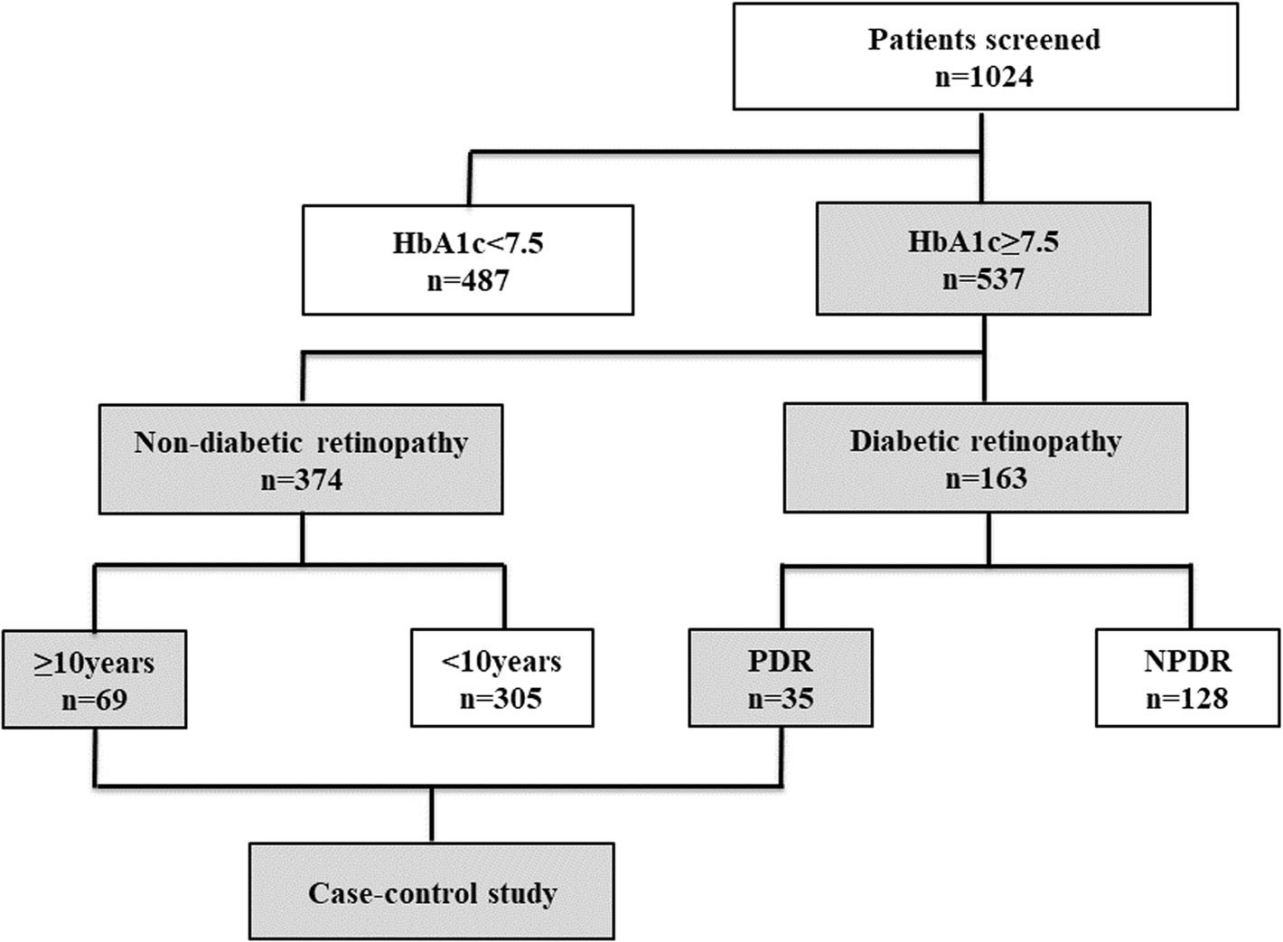
Inclusion and exclusion flowchart of the case-control study. NPDR, non-proliferative DR; NDR, non-DR; PDR, proliferative DR

Participants with type 2 diabetes (T2D) and extreme eye phenotype from our previous cohort study [13] performed a case-control study to find a plasma metabolite specific to eye damage. We chose patients with glycated hemoglobin A1c (HbA1c) ≥7.5% (58 mmol/mol) for eye phenotype screening. To match clinical parameters between case and control subjects, patients with sight-threatening PDR (case subjects) or those with a duration of diabetes of ≥10 years but without any degree of DR (non-diabetic retinopathy, NDR) (control subjects) were assigned (Fig. 1). Those individuals complicated with other eye diseases or with severe impairment of liver, kidney or heart function were excluded from selection.

### Baseline test

All of the participants’ medical histories were obtained and patients received a physical examination that included recording age, sex, duration, blood pressure, and body mass index (BMI). Patients underwent blood and urine laboratory tests that included fasting plasma glucose, total cholesterol, triglycerides, High density lipoprotein cholesterol (HDL-c), Low density lipoprotein cholesterol (LDL-c) serum creatinine, and HbA1c. K_2_EDTA tubes were used to collect blood samples. Tubes were centrifuged at 3000 g for 10 min (4 °C) to separate plasma from whole blood. Plasma aliquotswere stored at − 80 °C.

### Assessment of DR grading

Eye phenotype screening were conducted between April 2015 and July 2017 in Beijing, Tongren Hosppital, China. The presence of DR was diagnosed using digital retinal photographs (2 eyes × 2 fields) taken by using a TRC-NW7SF (Topcon Co. Tokyo, Japan) non-mydriatic camera at 45°. These photographs were subsequently examined independently by 2 qualified retinal photography graders in accordance with quality assurance protocols. The severity of DR was graded based upon the international clinical diabetic retinopathy and diabetic macular edema disease severity scale [14].

### Sample preparation

Before the analysis, frozen plasma was thawed and dissolved at 4 °C. We then added a mixture of acetonitrile/methanol (75:25 v/v, 300 μL) to the plasma (100 μL) in a 1.5-mL tube to precipitate proteins. This mixture was allowed to stand for 10 min after vortexing for 60 s, and then we centrifuged the samples at 12000 rpm for 10 min at 4 °C. The supernatant was transferred to a new eppendorf tube and then evaporated to dry in a speedvac concentrator. Afterward, the residues were re-suspended in the 100 μL mobile phase prior to LC-MS analysis. Quality control (QC) samples were prepared by mixing the same amount of serum from each sample and using the same procedures as the test samples to extract metabolites. One QC was inserted into every five samples regularly before and after operation.

### LC-MS (liquid chromatography-mass spectrometry) analysis

The ultra-performance liquid chromatography combined with quadrupole time-of-flight tandem mass spectrometry (UPLC Q-TOF MS) analysis was performed on Nexera X2 system (Shimadzu, Japan) coupled with a Triple TOF 5600 quadrupole-time-of-flight mass spectrometer (AB SCIEX, USA) as previous described [15]. In brief, liquid chromatography separation was performed on a ZORBAX Eclipse Plus C18 column (2.1 × 100 mm, 3.5 um, Agilent, USA) maintained at 45 °C. The injected sample volume was 10 μL for each run in the full loop injection mode, and the flow rate of the mobile phase was 0.5 mL/min. In RPLC mode, gradient elution was performed with the following solvent system: (A) 0.1% formic acid-water and (B) acetonitrile with 0.1% formic acid. The gradient started with 98% A, which decreased to 10% A in 13 min, holding at 10% A for 3 min; and then moved to 98% A immediately, holding at 98% A for 4 min. Mass spectrometric experiments were performed on a Triple TOF 5600+ orthogonally accelerated time-of-flight mass spectrometer (AB Sciex, USA) equipped with an electrospray ion source. We acquired data in positive- and negative-V-geometric modes for each chromatographic separation technique for LC-MS analysis. The capillary voltages were set to 2500 V and 3000 V, cone gas at 50 L/h, desolvation gas at 600 L/h, source temperature at 120 °C, and desolvation temperature at 500 °C. The scan range was from 50 to 1500 m/Z in the full scan mode, and data were collected in centroid mode. We used independent reference lock-mass ions via Analyst TF 1.6 and MarkerView 1.2.1 to ensure mass accuracy during data acquisition.

### Metabolites identification

**Fig. 2:**
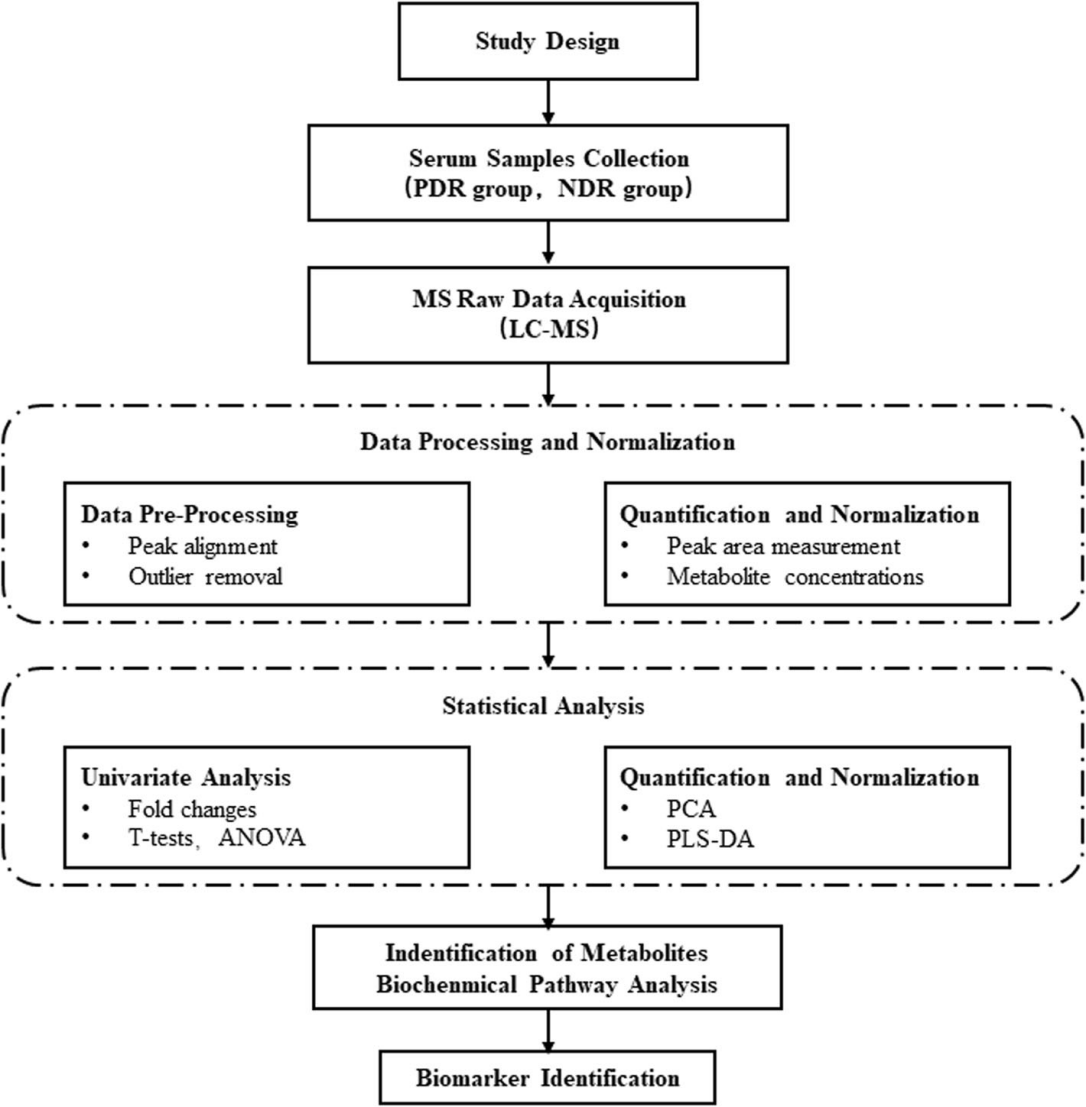
Workflow overview of the comprehensive analysis of metabolomics in patients with type-2 diabetes

By using MarkerView software to pre-process the raw UPLC Q-TOF MS data, such as retention time alignment, peak discrimination, filtering, alignment, matching, and identification, we generated a peak table with retention time (tR), m/z value and corresponding peak intensity. Multivariate analysis by MetaboAnalyst 4.0 program (http://www.metaboanalyst.ca/MetaboAnalyst/) including unsupervised principal component analysis (PCA) and supervised projections to latent structures-discriminant analysis (PLS-DA) were then used to identify differentiated metabolites between the 2 groups. The assigned metabolite ions were identified based on m/z and screened in the Human Metabolome Database HMDB (http://www.hmdb.ca/) [16]. The mass tolerance for the HMDB database search was set at 0.05 Da. We also considered the chromatographic retention behavior to reduce false-positive matches. An overview of workflow with respect to the comprehensive analysis of metabolomics in patients with type 2 diabetes is summarized in Fig. 2.

### Statistical analysis

Mann-Whitney U test were first performed to compare the PDR group with NDR groups. Multivariate analysis—including unsupervised principal component analysis (PCA) and supervised projections to latent structures-discriminant analysis (PLS-DA)—were used to determine the distributions and find the metabolic differences between 2 groups using MetaboAnalyst 4.0 (http://www.metaboanalyst.ca/MetaboAnalyst/). The PLS-DA models were cross-validated using a 10-fold method with unit variance scaling. The parameter R^2^ was used to evaluate the fitting condition for the PLS-DA models, and Q^2^ was used to assess predictive ability. Negative or very low Q^2^ values indicated that the differences between groups were not statistically significant. The PLS-DA model removes variation in the X matrix that is not correlated with the Y matrix. Thus, normally only 1 predictive component is used for the discrimination between 2 classes.

Comparisons of 2 groups related to the intensities of integrated regions were made by the 2-tailed Welch’s *t* test, which was performed using MetaboAnalyst 4.0, and a *p*-value < 0.05 was considered statistically significant. Volcano plots were calculated using a combination of fold-change and *t* tests, and the peaks that exhibited a statistically significant difference between 2 groups were used to perform multivariate pattern recognition. We then identified those peaks that showed consistent up-regulation or down-regulation; and the intensity data of these regions were used in box-plot analysis, hierarchical cluster analysis, and metabolic pathway analysis.

### Pathway analysis

In our study, the different chemical metabolites were evaluated using the Metaboanalyst web portal for pathway analysis and visualization (http://www.metaboanalyst.ca/). Additional pathway enrichment statistics were analyzed using Metabolite set enrichment analysis (MSEA) (http://www.msea.ca/MSEA/faces/Home.jsp). Pearson’s r correlation was calculated to evaluate relationships between/among the biomarkers (*P* < 0.05, impact > 0.01).

## Results

### Demographic and clinical information

Of the 1024 consecutive patients with T2D screened, 537 had a HbA1c > 7.5%. According to the inclusion and exclusion criteria of the case-control study, 21 sight-threatening PDR and 21 NDR with a diabetes duration ≥10 years were assigned as case and control subjects (Fig. 1). Characteristics of the participants in this study are summarized in Table 1. No significant differences were found between the groups with respect to age, gender, blood pressure, BMI, TG, TC, or LDL-c and HDL-c; however, there were differences in duration of diabetes, HbA1c, and albuminuria between PDR and NDR groups. Apparently, glucose in the NDR group was even more poorly controlled than in the PDR group. Diabetic patients with PDR were prone to albuminuria, which was associated with diabetic nephropathy.

**Table 1:** Baseline demographics in this study

	NDR	PDR	*P*-value
n	21	21	–
Gender (male/Female)	9/12	9/12	–
Age (years)	55 (50–58)	49 (46–56.5)	0.194
Diabetes duration(years)	15 (11.5–20)	11 (8–15.5)	0.014*
BMI (kg/m2)	26.9 (24.21–29.74)	25.8 (23.86–28.20)	0.473
SBP (mmHg)	129 (120–141.5)	132 (122–139)	0.537
DBP (mmHg)	78 (70.5–82.5)	74 (70–80.5)	0.348
Cr (umol/L)	60 (52.75–72.98)	75.5 (59.7–98.5)	0.041
TG (mmol/L)	1.87 (1.43–3.19)	1.97 (1.49–2.98)	0.843
TC (mmol/L)	4.69 (3.88–5.73)	4.91 (4.24–5.57)	0.792
LDL-c (mmol/L)	2.86 (2.19–3.54)	2.97 (2.43–3.55)	0.726
HDL-c (mmol/L)	0.96 (0.83–1.19)	1.03 (0.89–1.10)	0.770
HbA1c (%)	9.2 (8.35–10.10)	8.1 (7.57–8.75)	0.000**
Albuminuria	5	15	0.002†**
Microalbuminuria (20-200 ng/min)	4	8	
Macroalbuminuria (> 200 ng/min)	1	7	

Data are median (25th, 75th interval) unless otherwise indicated. Statistical analyses were by Mann-Whitney U test. † Pearson Chi-suuare test. *P < 0.05 and ** *P* < 0.01, NDR vs. PDR group

*Cr* creatinine, *SBP* systolic blood pressure, *DBP* diastolic blood pressure, *HDL-C* HDL cholesterol, *LDL-C* LDL cholesterol, *TC* total cholesterol, *TG* triglyceride

Metabolomics changes between NDR group and PDR group.

**Fig. 3:**
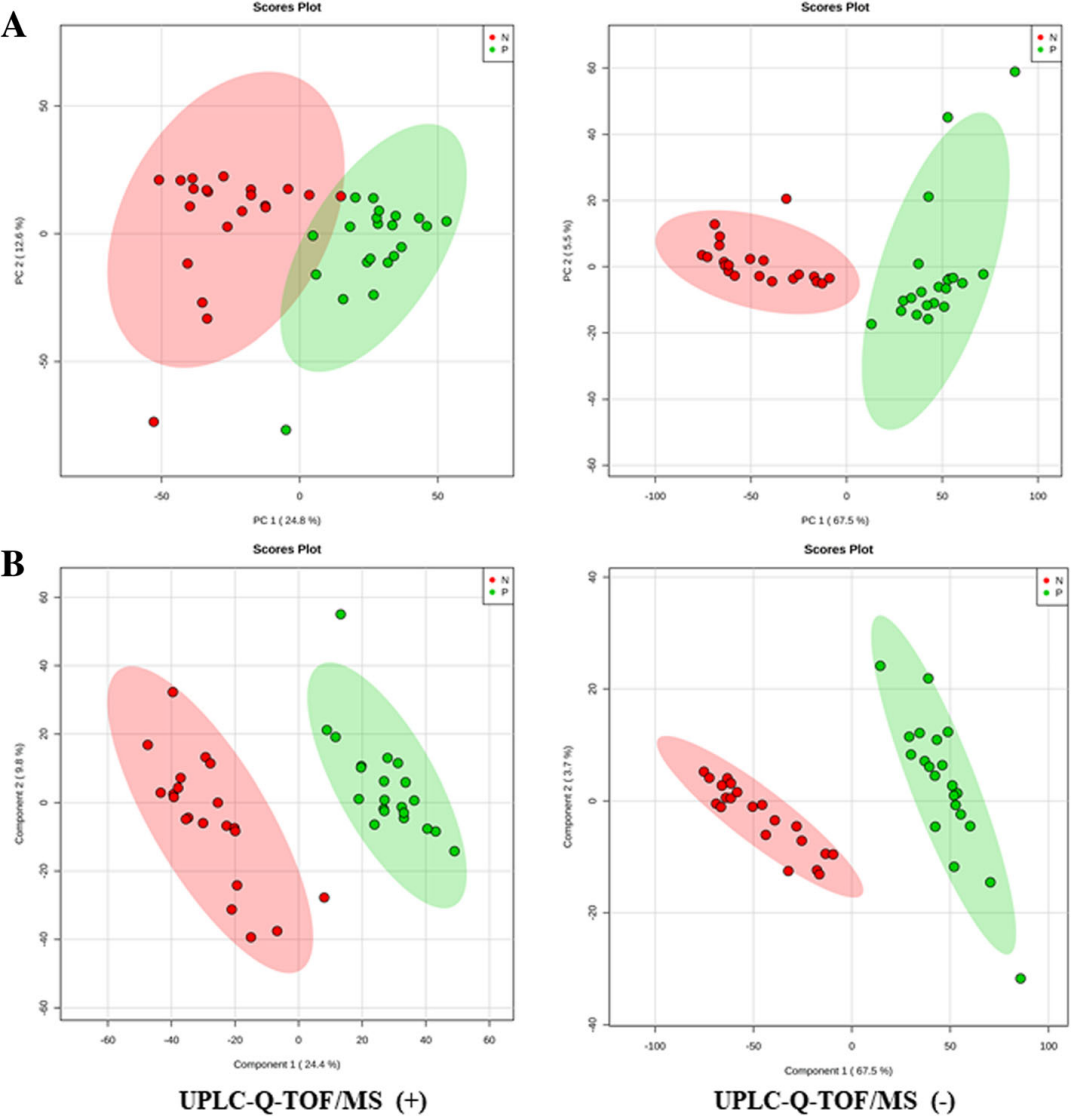
Score plots of the PCA and PLS-DA models. **a** Score plot of the PCA model for samples collected from 2 isolates of sample data; **b** the 2 groups were well separated in the PLS-DA score plot, indicating that they had markedly different metabolic characteristics

**Fig. 4:**
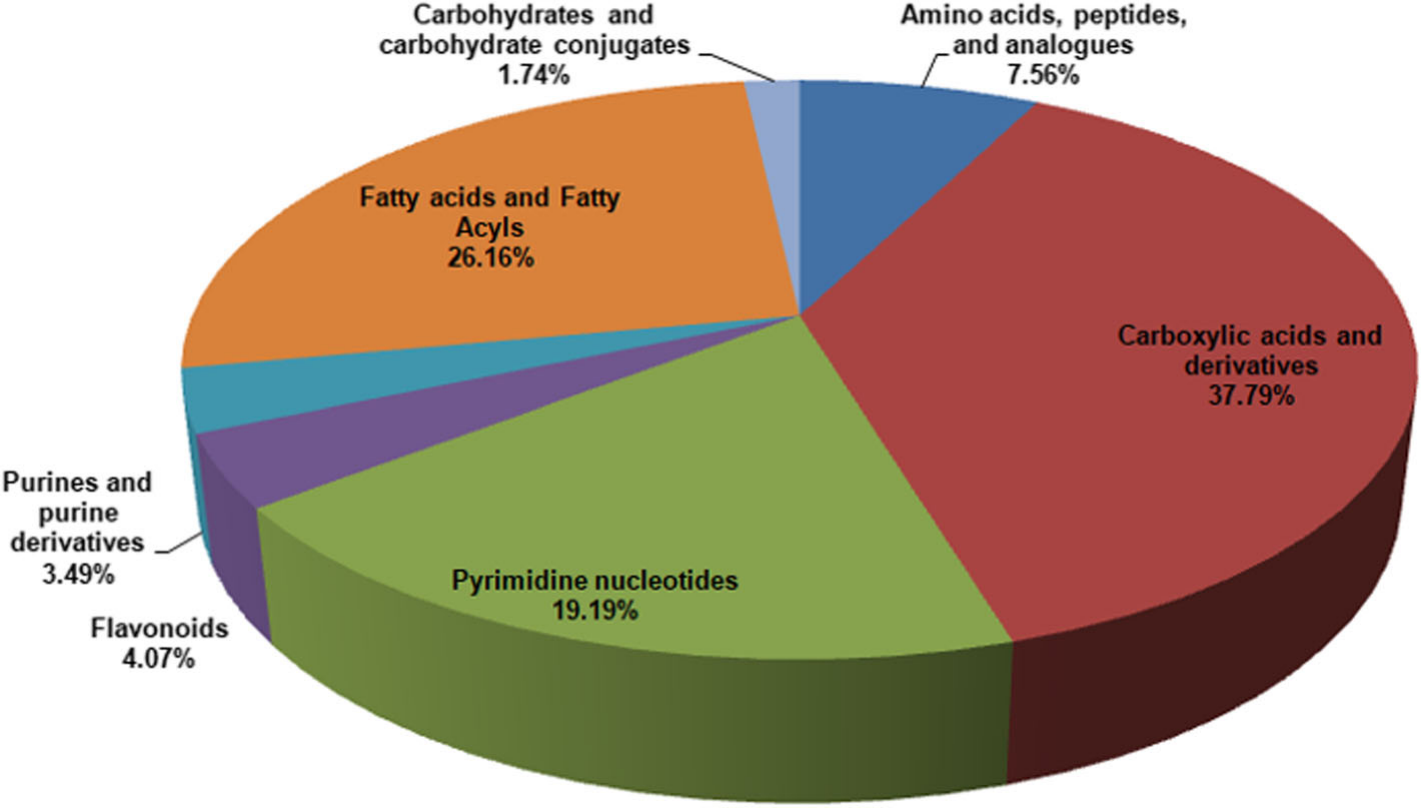
Metabolite classification analysis. Pie chart of differentially metabolomics showed that the 1027 metabolites mainly involved carboxylic acids and derivatives (37.79%), fatty acyls and fatty acid esters (26.16%), pyrimidine nucleotides (19.19%), amino acids, peptides, and analogues (7.56%) and others

A total of 7735 m/z were identified in both groups by LC-MS analysis. A score plot of the PCA model for samples collected from the 2 isolates of sample data is shown in Fig. 3a. To improve the separation of the 2 groups, we used PLS-DA to visualize the metabolic differences between them. The 2 groups were well separated in the PLS-DA score plot, indicating that they had markedly different metabolic characteristics (Fig. 3b). Considering the *p* values and fold-changes (FC), we drew a volcano map (Additional file 1). Based upon our criteria of *FC* > 1.5 or *FC* < 0.5 and *p* < 0.05, 1027 different metabolites were identified after HMDB database screening. These metabolites mainly included carboxylic acids and derivatives (37.79%); fatty acyls and fatty acid esters (26.16%); pyrimidine nucleotides (19.19%); amino acids, peptides, and analogues (7.56%); and other markers (Fig. 4).

### Models analysis

We constructed PCA and PLS-DA models to disclose the metabolic differences between classes, which were often confounded by the influences of diurnal variation, normal physiologic status, and other effects unrelated to responses of interest. All of the models were cross-validated by default using a 10-fold method, the validity of the models against over-fitting was assessed by the parameter R^2^, and the predictive ability was described by Q^2^. R^2^ and Q^2^ were 0.97 and 0.94 in PLS-DA model, respectively. Results showed great applicability of the PLS-DA model, and the established PLS-DA model was capable of differentiating case groups from control groups.

### Pathway analysis

**Fig. 5:**
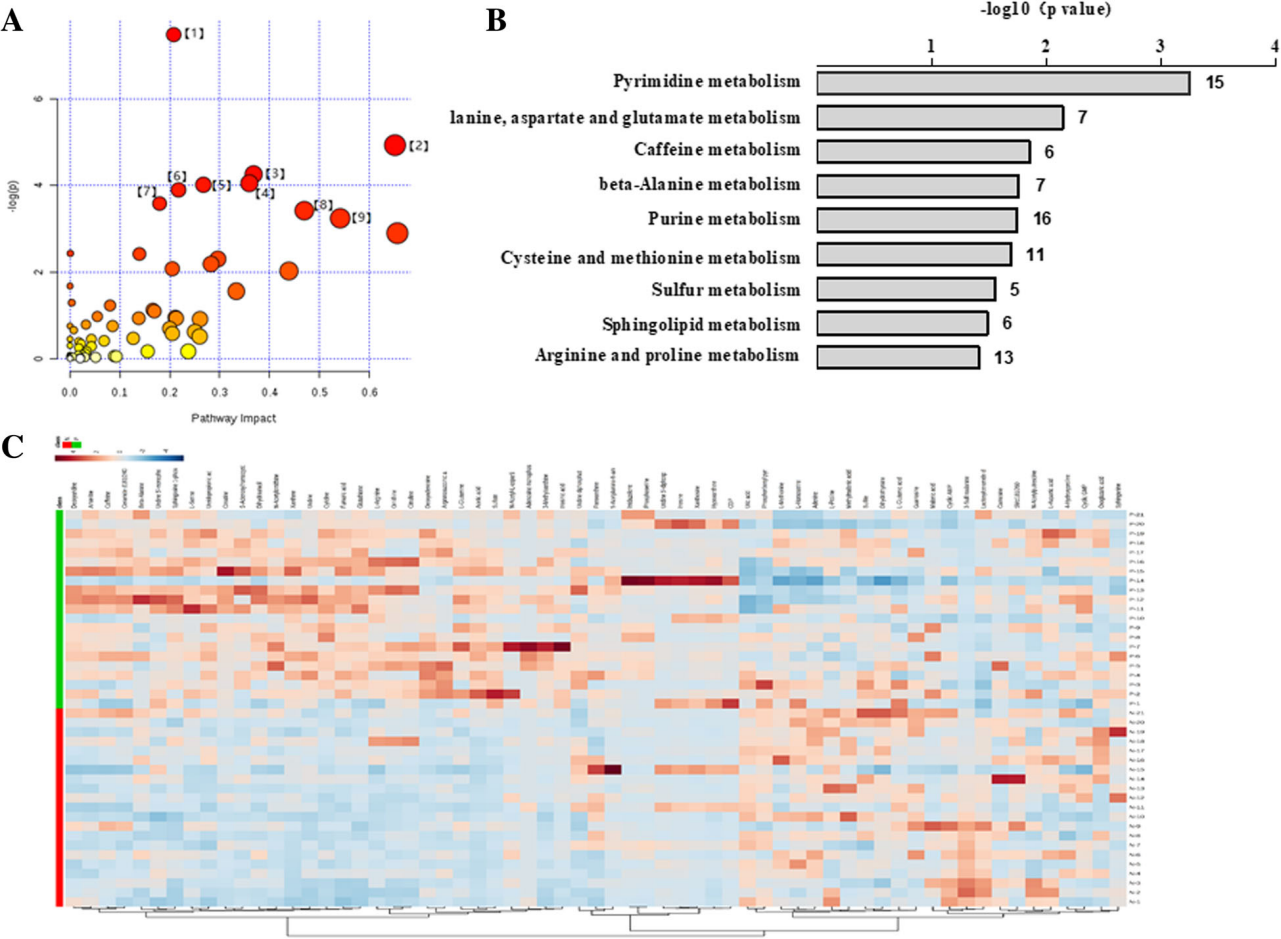
Non-targeted metabolomics pathway analysis. **a** Pathway enrichment analysis. The size and color of each circle was based on pathway impact value and *p*-value, respectively. **b** 9 pathways differ between the NDR and PDR group, particularly, in Pyrimidine metabolism, Alanine, aspartate and glutamate metabolism, Caffeine metabolism, beta-Alanine metabolism, Purine metabolism, Cysteine and methionine metabolism, Sulfur metabolism, Sphingolipid metabolism, Arginine and proline metabolism. **c** Heatmap of non-targeted plasma metabolomics show 63 compounds play important roles in 9 above-mentioned pathways indicated significantly different metabolites between NDR and PDR group

These 1027 metabolites were enriched in 62 KEGG PATHWAY Database (Additional file 1), of which 9 pathways were enriched significantly (*P* < 0.05, impact> 0.01) with impact factors of 0.21, 0.65, 0.37, 0.36, 0.27, 0.22, 0.18, 0.47, and 0.54, respectively (Fig. 5a, b). These pathways were pyrimidine metabolism; alanine, aspartate and glutamate metabolism; caffeine metabolism; beta-alanine metabolism; purine metabolism; cysteine and methionine metabolism; sulfur metabolism; sphingolipid metabolism; arginine and proline metabolism; 63 metabolites (Additional file 2) were identified in the aforementioned 9 pathways and a corresponding heatmap is shown in Fig. 5c.

### Biomarkers for PDR diagnosis

**Fig. 6:**
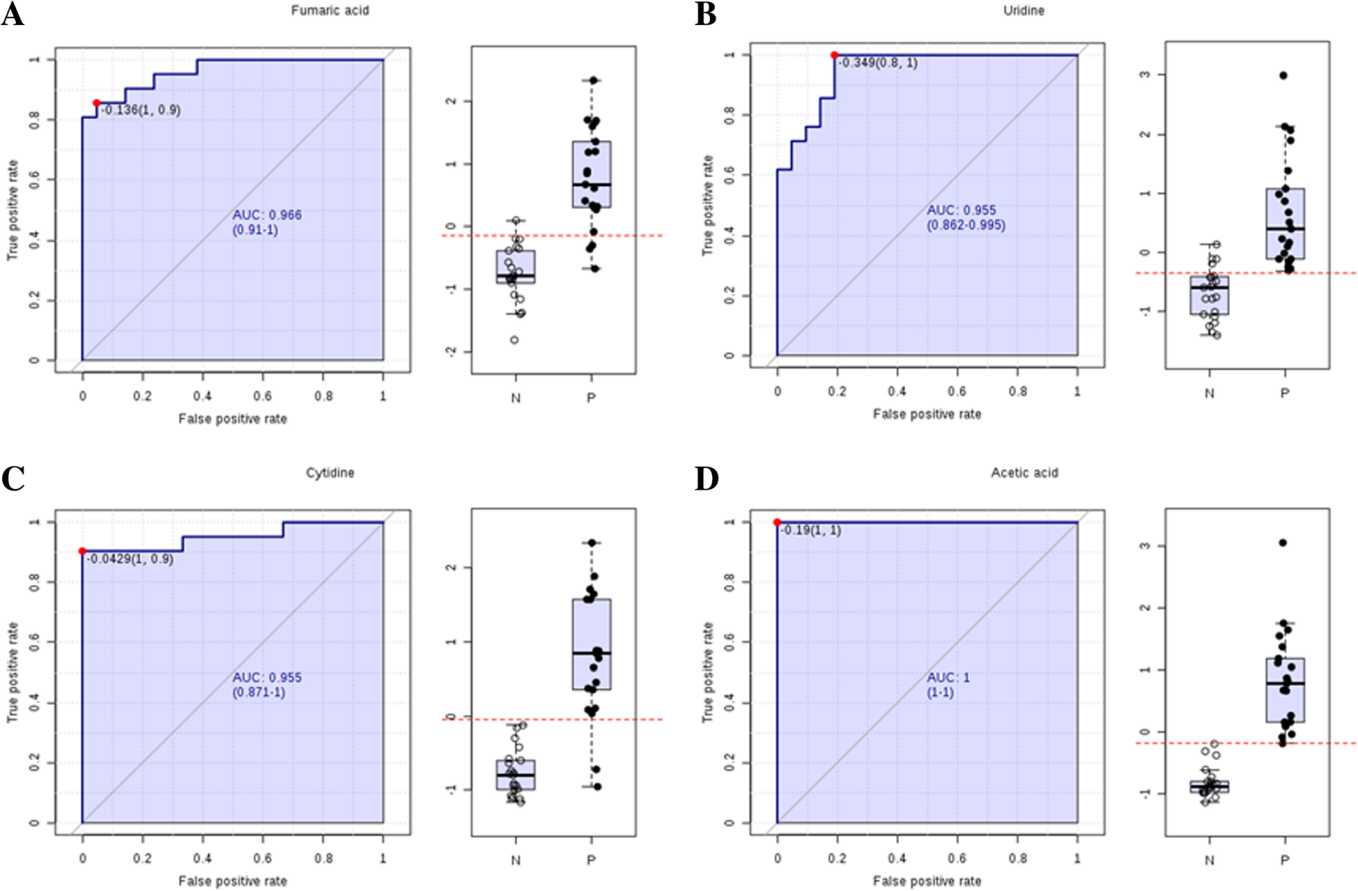
Receiver operating characteristic (ROC) curve analysis was performed to evaluate the use of metabolites as biomarkers for PDR. **a** The P value and AUC for fumaric acid were 7.90E-17 and 0.96, respectively; **b** the *P* value and AUC for uridine were 6.10E-16 and 0.95, respectively; **c** the P value and AUC for cytidine were 5.87E-12 and 0.95, respectively; and (**d**) the *P* value and AUC for acetic acid were 1.48E-13 and 1.0, respectively

We identified 63 compounds that contributed to the variations in the analyzed sample groups, and used them as potential markers to explain the variation between the NDR and PDR groups; In order to better screening metabolic compounds, we set a more strict selection criteria with *P* < 10E-05, an area under the curve (AUC) ≥0.95 and a VIP (Variable important in the projection) > 1 (Table 2). Seven metabolites were recognised afterward. The top 4 metabolites (*P* < 10E-11) were selected as candidate biomarkers for PDR, namely, fumaric acid, uridine, acetic acid, and cytidine. The *P* values of these biomarkers were 7.90E-17, 6.10E-16, 1.48E-13, and 5.87E-12, respectively; and the AUCs were 0.96, 0.95, 1.0, and 0.95, respectively (Fig. 6).

**Table 2:** Metabolite markers identified from discovery metabolomic profiling

Metabolite	Fold-Change (PDR/NDR)	Vip	Elements	Query_mass	Adduct	Trend	AUC	*P*-value
Fumaric acid	4.235	1.1898	HMDB0000134	114.9904	M-H	↑	0.96	7.90E-17
Uridine	4.2748	1.1764	HMDB0000296	243.0613	M-H	↑	0.95	6.10E-16
Acetic acid	2.1932	1.9268	HMDB0000042	60.9867	M + H	↑	1.0	1.48E-13
Cytidine	8.6763	1.0932	HMDB0000089	242.0796	M-H	↑	0.95	5.87E-12
3-Sulfinoalanine	0.57541	1.155	HMDB0000996	151.9927	M-H	↓	1	1.06E-11
3-Methylxanthine	4.5202	1.0224	HMDB0001886	165.0393	M-H	↑	0.96	1.02E-09
Sulfate	1.8943	1.3454	HMDB0001448	96.9718	M + H	↑	0.96	2.28E-05

## Discussion

Diabetic retinopathy, acknowledged as a chronic metabolic disease, is the mainly cause of blindness among working-age adults throughout the world [1]. Without treatment, it easily leads to vision loss and affect the quality of life. Thus, early detection and intervention are of great significance. The pathogenesis of diabetic retinopathy is related to hyperglycemia-induced persistent metabolic disorders, inflammation, oxidative stress, etc. [17]. It is an indisputable fact that proliferative diabetic retinopathy (PDR) also occurs in some patients whose blood glucose control is up to standard, suggesting other pathogenic factors contribute to this phenomenon [5]. Therefore, for better understanding the mechanism of the occurrence and development of the disease, it is essential to identify the biomarkers that lead to different phenotypes of diabetic retinopathy, which relates to the interplay between genetic and environmental factors. Recently, metabolomics has gained attention as an attractive tool for biomarker identification in metabolic disorders. In the present study, we used LC-MS-based metabolomic profiling to identify metabolic markers in diabetic patients with sight threaten proliferative diabetic retinopathy (PDR) in comparison to diabetics without retinopathy (NDR). Our aims were as follows: 1) to identify metabolic signatures that could distinguish patients with PDR from those with long duration but without DR, and 2) to identify the metabolic pathways that respond to DR.

We firstly reported that Fumaric acid, identified from metabolomic profiling, was associated with PDR and it might be a new biomarker and potential therapeutic target for DR. In our study, we observed high level of fumaric acid in the PDR group, and the AUC for detecting diabetic retinopathy was 0.96 (95% CI, 0.91–1). Fumaric acid is a small-molecule metabolite that recently be identified as an epigenetic modifier. Even though, its roles in DR have not yet been elucidated, some studies reported high levels of fumaric acid in many tumors and biofluids that surround tumors [18]. Studies suggested this metabolite might be involved in hypoxia in tumors, while the HIF transcription factor (HIF-α) was the major regulator in response to hypoxia [7, 19]. Moreover, Sciacovelli et al. [20] found excessive fumaric acid accumulation in renal cell cancer in response to an epigenetic change in micro RNA200. Previous findings suggested that both HIF-α [17, 21] and micro RNA200 [21, 22] played an important role in the formation of diabetic retinopathy. Retina is an organ with high metabolism and oxygen consumption in body, and is thought to be particularly susceptible to hyperglycemia-related oxidative stress [23–25]. Abnormal metabolism induced by hyperglycemia could also result in the overproduction of free radicals that can lead to oxidative stress and damage to tissues in and around retinal vessels [26]. Retinal microvascular dysfunction—resulting in retinal ischemia and hypoxia—could also aggravate oxidative stress, which is thought to be one of the crucial factors in the pathogenesis of DR [27]. These events suggested fumaric acid may play a role in tissue oxidative stress. Therefore, we ultimately hypothesized that fumaric acid was associated with diabetic retinopathy, and that it might be a new biomarker and potential therapeutic target for DR. Further studies should be carried out to confirm this result and to investigate the mechanism of fumaric acid in DR development.

An earlier metabonomic study demonstrated an association of pyrimidine metabolism with the development of non-proliferative DR using gas chromatography-mass spectrometry (GC-MS) [28]. These investigators found higher levels of cytidine (*P* = 0.001) and thymidine (*P* = 0.001) in plasma samples from T2D patients with early stage of DR compared to those without DR, and cytidine had the highest AUC (0.849 ± 0.048). Pyrimidines are vital biomolecules that participate in a wide range of biological functions, including syntheses of DNA, RNA, lipids, and carbohydrates [29] and pyrimidine derivatives are already being used as antidiabetic medications [30]. In our study, we observed increased levels of cytidine in PDR patients, which was similar to previous findings. Cytidine, a pyrimidine molecule, is considered as the precursor of the cytidine triphosphate (CTP), which influences metabolism of phospha-tidylcholine (PC) and phosphatidylethanolamine (PE) biosynthesis. Besides that, alteration of cytidine could induce the abnormality of the salvage pathway of pyrimidine nucleotide, followed by the dysfunction of phospholipid [28]. Previous studies reported the concentration of phospholipids decreased with the development of diabetic microvascular complications [31]. Therefore, we suppose that cytidine may serve as a potential biomarker for the diagnosis of diabetic retinopathy and evaluation of treatment. In current study, the concentration of uridine also changed significantly in the PDR group. Uridine is produced from cytidine by cytidine deaminase [32]. As a member of pyrimidine, it is also a promoting metabolic biomarker of diabetic retinopathy which is related to pyrimidine metabolism.

There are few metabolomics studies of diabetic retinopathy in the literature. Two previous studies concerning vitreous samples from patients with PDR and control patients without diabetes have suggested a dysregulation in several biochemical pathways, including the arginine to proline pathway [33], polyol pathway, and ascorbic acidic pathways [12]. Another two studies were performed on plasma samples from patients with NPDR to study the metabolic signature of DR. Chen et al. [6] also demonstrated significant enrichment of the pentose phosphate pathway. Paris et al. [33] used LC-MS to generate and validate the metabolomic profile of vitreous samples, and pathway enrichment analysis revealed that arginine metabolism was the pathway perturbed in the DR group. Our study supports a dysregulation of pathways, including arginine and proline metabolism. Elevated levels of arginine and ornithine were observed in patients with PDR, potentially implicating a compromised Mueller glial cell metabolism in the disruption of neurovascular crosstalk within the retina and progression of diabetic retinopathy [34, 35].

## Limitations

The relatively small sample size of our study might be insufficient to make conclusive statements regarding metabolic status in DR patients. Larger cohorts of diabetic patients need to be enrolled and metabolomic analyses performed to confirm our findings. The exact mechanism(s) underlying this disease remains elusive and requires further study.

## Conclusion

Herein, we generated a metabolomic profile between PDR and NDR groups in plasma of diabetic patients, and we found fumaric acid, cytidine, uridine, and acetic acid to be correlated with DR. Fumaric acid was firstly reported as a novel metabolite marker with no prior reports of association with diabetes or DR. Our study also replicated previous findings showing significant impairment in the metabolism of pyrimidines, arginine, and proline, which was identified as metabolic dysregulations associated with DR. Further research is required to replicate these findings and determine longitudinal associations with disease.

## Additional files

Additional file 1: KEGG PATHWAY Database. (XLSX 12 kb)

Additional file 2: Metabolites Database. (XLSX 13 kb)

## Abbreviations

AUC: Area under the curve
BMI: Body mass index
DBP: Diastolic blood pressure
DR: Diabetic retinopathy
HbA1c: Glycated hemoglobin A1c
HDL-C: High density lipoprotein cholesterol
LC-MS: Liquid chromatography-mass spectrometry
LDL-C: Low density lipoprotein cholesterol
NDR: Non - diabetic retinopathy
NPDR: Non - proliferative diabetic retinopathy
PCA: Principal component analysis
PDR: Proliferative diabetic retinopathy
PLS-DA: Projections to latent structures-discriminant analysis
SBP: Systolic blood pressure
T2D: Type 2 diabetes
TC: Total cholesterol
TG: Triglyceride
VIP: Variable important in the projection
